# Magnetosheath jets at Mars

**DOI:** 10.1126/sciadv.adg5703

**Published:** 2023-06-02

**Authors:** Herbert Gunell, Maria Hamrin, Sara Nesbit-Östman, Eva Krämer, Hans Nilsson

**Affiliations:** ^1^ Department of Physics, Umeå University, 901 87 Umeå, Sweden.; ^2^ Swedish Institute of Space Physics, Box 812, 981 28 Kiruna, Sweden.

## Abstract

Plasma entities, known as magnetosheath jets, with higher dynamic pressure than the surrounding plasma, are often seen at Earth. They generate waves and contribute to energy transfer in the magnetosheath. Affecting the magnetopause, they cause surface waves and transfer energy into the magnetosphere, causing throat auroras and magnetic signatures detectable on the ground. We show that jets exist also beyond Earth’s environment in the magnetosheath of Mars, using data obtained by the MAVEN spacecraft. Thus, jets can be created also at Mars, which differs from Earth by its smaller bow shock, and they are associated with an increased level of magnetic field fluctuations. Jets couple large and small scales in magnetosheaths in the solar system and can play a similar part in astrophysical plasmas.

## Teaser

Outside Earth's space environment, magnetosheath jets were observed at Mars by the MAVEN spacecraft

## INTRODUCTION

Magnetosheath jets are transient enhancements of the dynamic pressure in the magnetosheath plasma. The dynamic pressure enhancement can be due to an increased density, velocity, or both, and these jets are frequently observed in the magnetosheath of Earth (*1*). Jets, moving across magnetic fields in a vacuum (*2*) and through background plasmas (*3*), were first studied in laboratory experiments, where they were called plasmoids. The topic is also related to ion beams in fusion plasmas (*4*).

At Earth, magnetosheath jets were first reported in the late 1990s (*5*), and a substantial body of work has emerged in recent years (*6*–*16*). The typical scale size of the jets is 0.1 *R*_E_, where *R*_E_ is the radius of Earth, but sizes up to a few *R*_E_ have been observed (*13*). Jets have been seen to generate waves in the magnetosheath (*8*, *9*), to cause surface waves on the magnetopause (*10*, *15*, *17*), and to emit Alfvén waves that can be detected by ground-based magnetometers (*7*, *14*). They have also been associated with throat aurora (*18*). Structures similar to magnetosheath jets have been observed also upstream of the bow shock in the solar wind (*19*), but the majority of the jets are thought to be created at the bow shock (*20*). Several formation mechanisms have been suggested: solar wind interaction with ripples on the bow shock (*6*), which in turn could be associated with Short Large Amplitude Magnetic Structures (SLAMS) (*19*); hot flow anomalies (*21*); and discontinuities in the solar wind interacting with the bow shock (*22*) and bow shock reformation (*16*). Thus, jets are an integral part of the coupling of the large scale of the bow shock down to the small scale of waves in the magnetosheath. They contribute to the mix of different plasma populations in the magnetosheath and to the transfer of solar wind energy to wave energy in the magnetosheath, at the magnetopause, and, ultimately, down to the ionosphere as in the case of throat auroras.

**Fig. 1.**
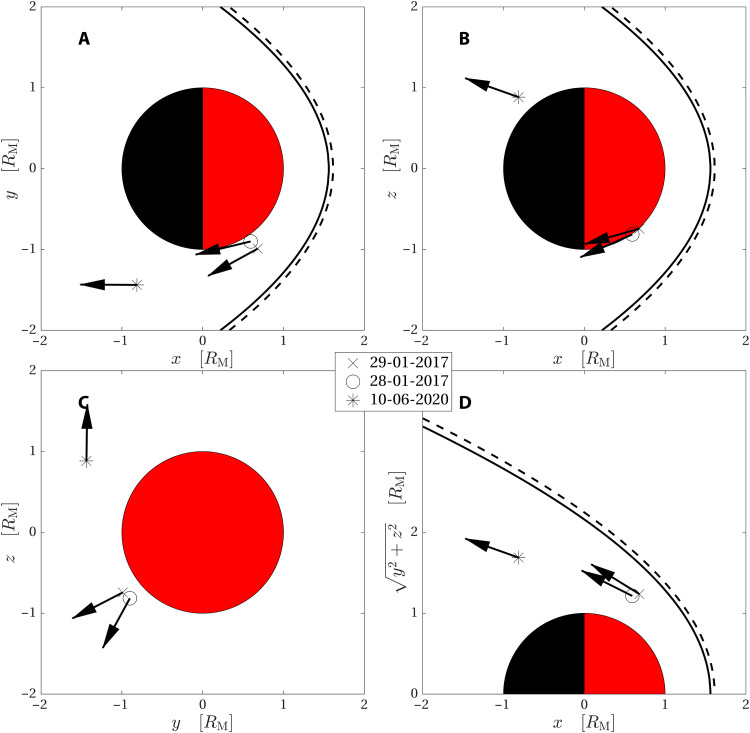
Observation location. Positions of the MAVEN spacecraft in Mars Solar Orbital (MSO) coordinates during the three observations. Spacecraft positions projected onto (**A**) the *x*-*y*, (**B**) the *x*-*z*, and (**C**) the *y*-*z* plane. (**D**) Spacecraft positions in a cylindrical coordinate system, where the vertical axis represents the distance to the MSO *x* axis. The arrows show the direction of the velocity component in the plane of each panel. The velocity is normalized so that all arrows have the same length. The component in the *y*-*z* plane (C) is substantially smaller than the other components. The dashed and solid lines show a model bow shock (*27*) for two different parameter sets. The spacecraft moves about 100 to 200 km during a jet observation, which is smaller than the symbol that marks the positions in the figure.

Until now, Earth is the only place where magnetosheath jets have been observed. However, all planets have bow shocks and so have comets if their gas production rate is high enough (*23*), and shocks are ubiquitous in astrophysics (*24*). Understanding how jets are formed will be aided by comparing results from Earth with other solar system objects. At Mercury, structures with a decreased magnetic field, i.e., the opposite of SLAMS, were found in the foreshock (*25*), but the resolution of the data did not allow a direct jet observation. Mars, because it is unmagnetized, has an induced magnetosphere, which in comparison with Earth’s magnetosphere, constitutes a substantially smaller obstacle to the solar wind. Therefore also, the bow shock of Mars is smaller and closer to the planet than the corresponding boundary at Earth; see Fig. 1. In this work, we use data obtained by the MAVEN spacecraft (*26*) to show that jets exist also in the magnetosheath of Mars.

## RESULTS AND DISCUSSION

We present three examples of jets observed by the MAVEN spacecraft on 28 and 29 January 2017 and on 10 June 2020. The spacecraft positions at the times of observation are shown in Fig. 1, both projected onto the principal planes of the Mars Solar Orbital (MSO) coordinate system and in cylindrical coordinates. The directions of the in-plane velocity vectors are shown as arrows, normalized so that all arrows have the same length. The dashed and solid lines show a model bow shock (*27*) for two different parameter sets: *n*_SW_ = 1.2 cm^−3^, *v*_SW_ = 610 km s^−1^ (solid line) and *n*_SW_ = 2 cm^−3^, *v*_SW_ = 300 km s^−1^ (dashed line). The solid line corresponds, approximately, to the solar wind parameters observed when the spacecraft was in the solar wind on 28 January 2017 and the same line can also represent the situation on 29 January 2017 as the parameters on that day would not produce a notable difference in the position of the model bow shock. On 10 June 2020, the spacecraft did not pass into the solar wind and the parameters used for that day were estimated from measurements in the magnetosheath during that day.

**Fig. 2.**
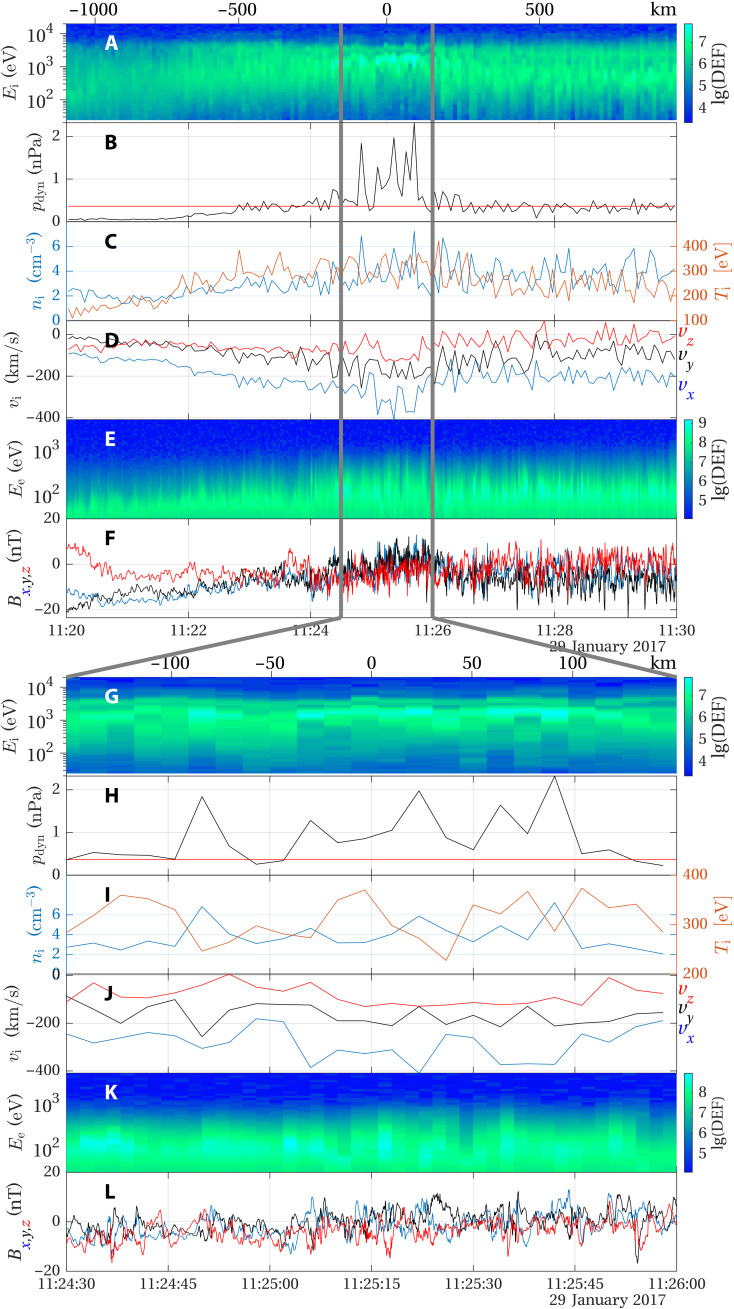
MAVEN observations on 29 January 2017. The displayed quantities are (**A**) the differential energy flux (DEF) of the ions as measured by the Solar Wind Ion Analyzer (SWIA) instrument; (**B**) the dynamic pressure of the ions, the red line represents the average dynamic pressure during the 10-min interval shown; (**C**) the ion density and temperature measured by the SWIA instrument; (**D**) the ion velocity components in MSO coordinates; (**E**) the electron DEF measured by the Solar Wind Electron Analyzer instrument; and (**F**) magnetic field components in MSO coordinates measured by the magnetometer investigation instrument. (**G** to **L**) show the data between the gray lines in (A) to (F). The scales on top of (A) and (G) show the distance traveled by the spacecraft from the center of the interval between the two gray lines, approximately at the center of the jet observation.

Figure 2 shows ion, electron, and magnetic field data from 29 January 2017. Panels (A) to (F) of Fig. 2 span a 10-min interval around the jet and panels (G) to (L) of Fig. 2 show a close-up of the jet itself and its immediate surroundings. The jet is identified as the period with several peaks in the dynamic pressure around 11:25:15, between the gray lines in Fig. 2B. The horizontal red line at *p*_dyn_ = 0.36 nPa shows the mean dynamic pressure over the 10-min interval. At Earth, several different selection criteria have been used [see (*11*) for a summary]. We will use a modified version of the criterion by Archer and Horbury (*28*), requiring that the jet dynamic pressure is larger than the mean value by a factor of 2, but since the magnetosheath of Mars is smaller than that of Earth, using a 10- instead of a 20-min average. All the peaks in Fig. 2H are above 2 < *p*_dyn_ > = 0.72 nPa, satisfying the criterion. The dynamic pressure drops below the threshold around 11:25:00, and strictly applying the criterion, what is seen before and after that time would be two separate jets. However, as they appear so close together, we consider them as being part of the same entity.

**Fig. 3.**
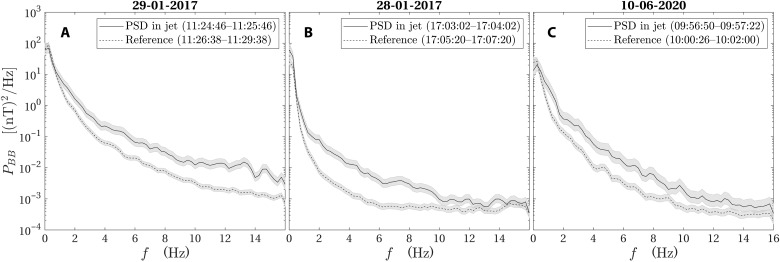
Power spectral densities. The sum of the power spectral densities (PSDs) of the three magnetic field components for the jets observed on (**A**) 29 January 2017, (**B**) 28 January 2017, and (**C**) 10 June 2020. The solid lines show the PSD inside the jet and the dashed lines show the same quantity during a reference interval near but outside each jet. The gray areas mark the 95% confidence intervals of each PSD estimate.

The ion spectrum (Fig. 2, A and G) shows a majority of protons and, separated in energy, an alpha particle population above 10^3^ eV/e. The scale length for thermalization after passing the bow shock is longer for the alpha particles than the protons due to the larger gyro radius of the alpha particles. The enhanced dynamic pressure of the jet is associated with both an increased density (Fig. 2, C and I) and a more negative *v_x_* velocity component. This means that the jet is moving more toward the anti-sunward direction than the flow of the surrounding magnetosheath. The temperature and density show a negative correlation (Fig. 2, C and I), and the electron spectrum (Fig. 2, E and K) shows no notable difference between the jet itself and the surrounding plasma. The magnetic field (Fig. 2F) was stronger at the beginning of the period shown, until approximately 11:23, as the spacecraft moved up from lower altitudes. Figure 2F also shows that there are stronger fluctuations in the magnetic field inside the jet than in the magnetosheath proper. The enhanced fluctuation level is also seen in Fig. 3A, which compares the power spectral density (PSD) inside the jet (11:24:46 to 11:25:46) to a reference period after the jet (11:26:38 to 11:29:38).

**Fig. 4.**
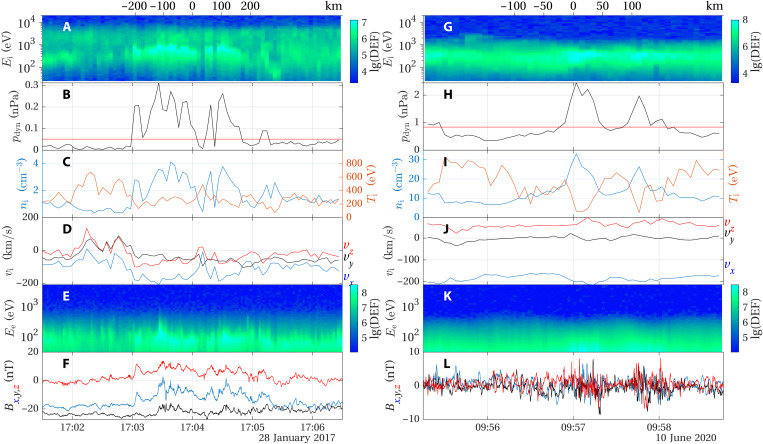
Observations on 28 January 2017 and 10 June 2020. (**A** and **G**) Ion DEF; (**B** and **H**) dynamic pressure of the ions (black line), 10-min average (red line); (**C** and **I**) ion density and temperature measured; (**D** and **J**) ion velocity components in MSO coordinates; (**E** and **K**) electron DEF; and (**F** and **L**) magnetic field components in MSO coordinates. Both a close-up of the jets themselves and the complete 10-min interval for these two cases are shown in figs. S1 and S2. The scales on top of (A) and (G) show the distance traveled by the spacecraft from the center of the interval shown, approximately at the center of the jet observation.

Two more examples of jet observations are shown in Fig. 4, where panels (A) to (F) correspond to an observation on 28 January 2017, and the observation in panels (G) to (L) took place on 10 June 2020. The position where the jet was observed on 28 January 2018 was closer to the planet than the jet on 29 January 2019 as is seen in Fig. 1, and the stronger magnetic field (Fig. 4F) shows that the spacecraft encountered the jet in a region characterized by magnetic pileup. The increase in dynamic pressure also for this jet is associated with increases in both density (Fig. 4C) and speed (Fig. 4D). The solar wind speed, estimated at the time of the nearest bow shock crossing, is similar in the two cases: 550 km s^−1^ on 29 January 2017 and 610 km s^−1^ on 28 January 2017. However, the jet observed on 28 January had slowed down more at the point of observation—seen as *v_x_* ≳ −200 km s^−1^ (Fig. 4D) compared to *v_x_* ≳ −400 km s^−1^ in Fig. 2J and also by the lower proton energy in Fig. 4A than in Fig. 2G. Similarly, at Earth, jets have been seen to slow down more the longer they travel through the magnetosheath (*11*). As in the previous case, the electron spectrum (Fig. 4E) shows higher fluxes when the density is higher but is otherwise relatively uneventful.

The jet observation on 10 June 2020 happened at a position farther downstream than the other two (Fig. 1) and also at a higher altitude. In Fig. 4H, two pulses of increased dynamic pressure are seen. As a result of the longer distance to the bow shock, the alpha particles have had time to mix with the protons and they cannot be seen as a distinct population in Fig. 4G. The enhanced dynamic pressure (Fig. 4H) is dominated by an increased density (Fig. 4I). The magnetic field fluctuations have a higher amplitude inside than outside the jets as the time series in Fig. 4L shows. This is true for all three examples as seen in the PSD plots in Fig. 3, indicating that the jets excite waves as they move through the magnetosheath. Potentially, jets could also be caused by steepening of waves, and, at Earth, jets have been seen together with mirror mode waves (*29*). In the present case, waves are not likely to cause the jets, as the waves we have observed are in the 1- to 16-Hz frequency range. Thus, the wave period is much shorter than the duration of the jet.

For the cases on 28 and 29 January 2017, the angle θ_Bn_ between the bow shock normal and the interplanetary magnetic field was in the 50^β^ to 70^β^ range when estimated both using coplanarity methods (*30*) and an empirical model of the bow shock (*27*). This indicates that the jets were observed downstream of the quasi-perpendicular bow shock on those dates. The bow shock is considered quasi-perpendicular for θ_Bn_ > 45^β^ and quasi-parallel for θ_Bn_ < 45^β^. At Earth, jets exist behind both the parallel and perpendicular shocks, but they are approximately nine times more common downstream of the quasi-parallel than the quasi-perpendicular shock (*31*). For the observation on 10 June 2020, no estimate can be made because the spacecraft’s orbit did not cross the bow shock on that date. The three cases in the present study are not enough to tell whether jets are more frequent at the parallel or the perpendicular bow shock at Mars.

Although this is a single-spacecraft study, a simple estimate can be made of the size of the jets. Comparing the jet size to the size of the magnetosheath, we can draw conclusions about the shape of the jet and where it comes from. The size of the jet in the direction of the ion velocity is in the range of 4000 to 18,000 km. This is much larger than the 100 to 200 km traversed by the spacecraft during the observation. The length of 4000 to 18,000 km is also of the order of or larger than the distance from the bow shock to the observation point. Thus, the jets should be seen rather as beams being continuously generated at the bow shock, on time scales longer than the ion transit time through the magnetosheath than as short ball-like structures passing by the spacecraft.

At Earth, jets are known to be both ubiquitous and geoeffective, and they contribute to wave generation and energy transfer in the magnetosheath. We have shown that magnetosheath jets exist beyond Earth’s environment, and even with the difference in the scale and character of the martian magnetosphere, they play a similar role in the martian magnetosheath. A quantitative estimate of the geoeffectiveness of magnetosheath jets at Mars requires a larger statistical study covering all regions of space around the planet as well as different solar wind conditions. This is planned in future research.

## MATERIALS AND METHODS

The ion spectra and moments were measured using the Solar Wind Ion Analyzer (SWIA) (*32*). The electron spectrum was measured by The MAVEN Solar Wind Electron Analyzer (*33*). Last, the magnetic field was measured by the magnetometer investigation (*34*). Throughout this work, we use the MSO coordinate system, where the origin is at the center of Mars, the *x* axis points toward the Sun, the *y* axis is in the orbital plane of Mars directed opposite to the orbital velocity of the planet, and the *z* axis closes the right-handed system. The jet velocity used in Fig. 1 was found by averaging the SWIA velocity moment during the intervals shown in Table 1. The same intervals were used to compute the PSDs in Fig. 3. The velocities in MSO coordinates are also shown in Table 1. The size of the jet in the direction of the ion velocity was estimated as the product of the ion speed and jet duration.

**Table 1.** Jet intervals and mean ion velocities in MSO coordinates during those intervals.

Date	Start	End	*v_x_*	*v_y_*	*v_z_*
29 January 2017	11:24:46	11:25:46	−306 km/s	−168 km/s	−87 km/s
28 January 2017	17:03:02	17:04:02	−175 km/s	−42 km/s	−77 km/s
10 June 2020	09:56:50	09:57:22	−195 km/s	1 km/s	69 km/s

The solar wind parameters used to compute the bow shock position for the cases in January 2017 were taken during the closest time to the jet observation MAVEN was in the solar wind. For 10 June 2020, MAVEN was not in the solar wind, and the parameters used were retrieved at 05:46, when the proton spectrum was relatively narrow, indicating that the spacecraft was close to the bow shock. This was still in the magnetosheath and 4 hours before the jet observation. However, we see from Fig. 1 that the difference between the bow shock positions for the two curves shown in the figure is small in comparison to the width of the magnetosheath and the size of the planet.

We have verified that the bulk of the jet flux is within the field of view (FOV) of the SWIA instrument by constructing FOV maps as shown in fig. S3. For the three cases analyzed here, the peak of the flux is in the interior of the FOV. The fluxes in bins at the edges of the FOV are approximately one order of magnitude below the peak value. In fig. S3, we have ignored the energy dependence of the elevation angle θ and plotted the flux for all energy on the θ scale for the highest energy because, to test whether the ions are in the FOV, it is the bins that are of interest rather than the angles of incidence. The moments are not corrected for the spacecraft potential. However, the correction would be too small to influence the results, as the spacecraft potential is much lower than the proton energy in the magnetosheath.

To select the events, we examined all data from December 2016, January 2017, and June 2020. Jet candidates were seen on approximately half of the days examined. We then selected three cases that meet the jet criterion described in the text, and where the bulk of the flux was in the FOV. Also, these three were chosen to exemplify jets in different environments. The two from January 2017 are on the dayside, with the one from 28 January in a stronger magnetic field than the jet observed on 29 January. The case observed on 10 June 2020 was recorded further downstream of the terminator.
