# Supplementary material for: Magnetosheath jets at Mars
Source: Sci Adv. 2023 Jun 2;9(22):eadg5703. doi: 10.1126/sciadv.adg5703 (PMC10413640; doi:10.1126/sciadv.adg5703)
Supplement: Supplementary file 1 — Figs. S1 to S3 [file sciadv.adg5703_sm.pdf]

Science Advances
AAAS

Supplementary Materials for

Magnetosheath jets at Mars

Herbert Gunell *et al.*

Corresponding author: Herbert Gunell, herbert.gunell@physics.org

Sci. Adv. **9**, eadg5703 (2023)
DOI: 10.1126/sciadv.adg5703

This PDF file includes:

Figs. S1 to S3

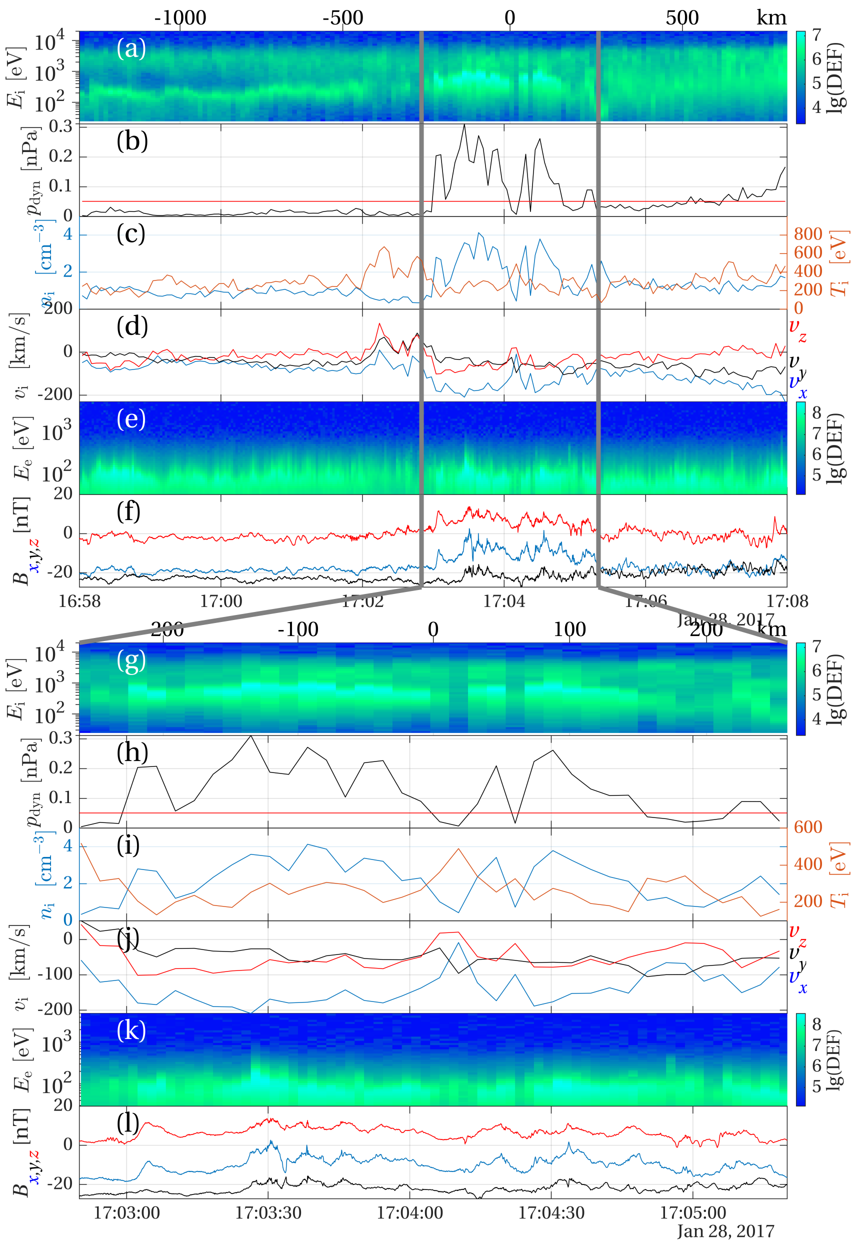

Figure S1: **MAVEN observations on 28 January 2017.** The displayed quantities are (**a**) the differential energy flux of the ions as measured by the SWIA instrument; (**b**) the dynamic pressure of the ions, the red line represents the average dynamic pressure during the ten minute interval shown; (**c**) ion density and temperature measured by the SWIA instrument; (**d**) the ion velocity components in MSO coordinates; (**e**) electron differential energy flux measured by the SWEA instrument; and (**f**) magnetic field components in MSO coordinates measured by the MAG instrument. Panels (**g**)–(**l**) show the data between the grey lines in panels (**a**)–(**f**). The scales on top of panels (a) and (g) shows the distance travelled by the spacecraft from the centre of the interval between the two grey lines, approximately at the centre of the jet observation.

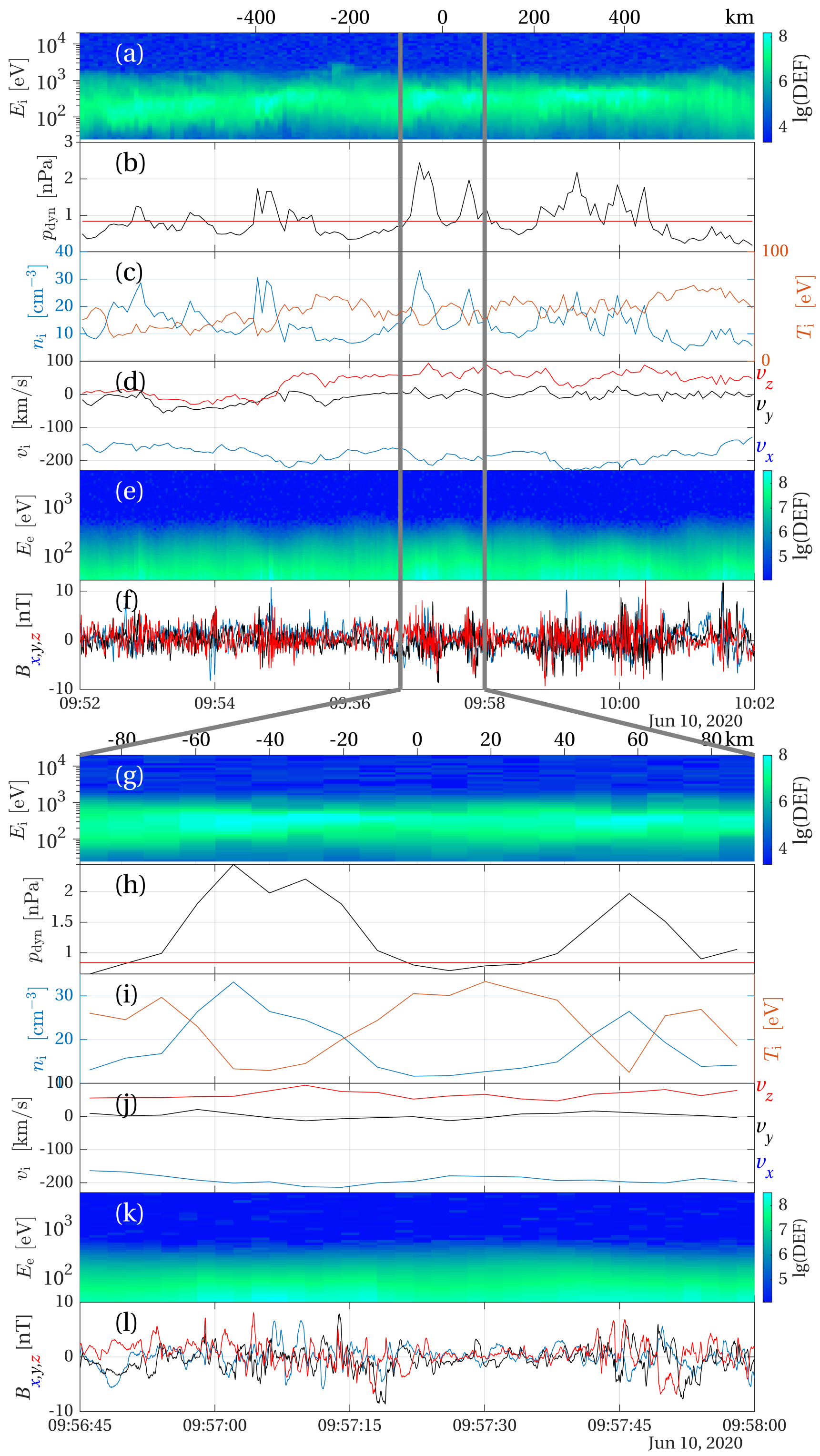

Figure S2: **MAVEN observations on 10 June 2020.** The displayed quantities are (**a**) the differential energy flux of the ions as measured by the SWIA instrument; (**b**) the dynamic pressure of the ions, the red line represents the average dynamic pressure during the ten minute interval shown; (**c**) ion density and temperature measured by the SWIA instrument; (**d**) the ion velocity components in MSO coordinates; (**e**) electron differential energy flux measured by the SWEA instrument; and (**f**) magnetic field components in MSO coordinates measured by the MAG instrument. Panels (**g**)–(**l**) show the data between the grey lines in panels (**a**)–(**f**). The scales on top of panels (a) and (g) shows the distance travelled by the spacecraft from the centre of the interval between the two grey lines, approximately at the centre of the jet observation.

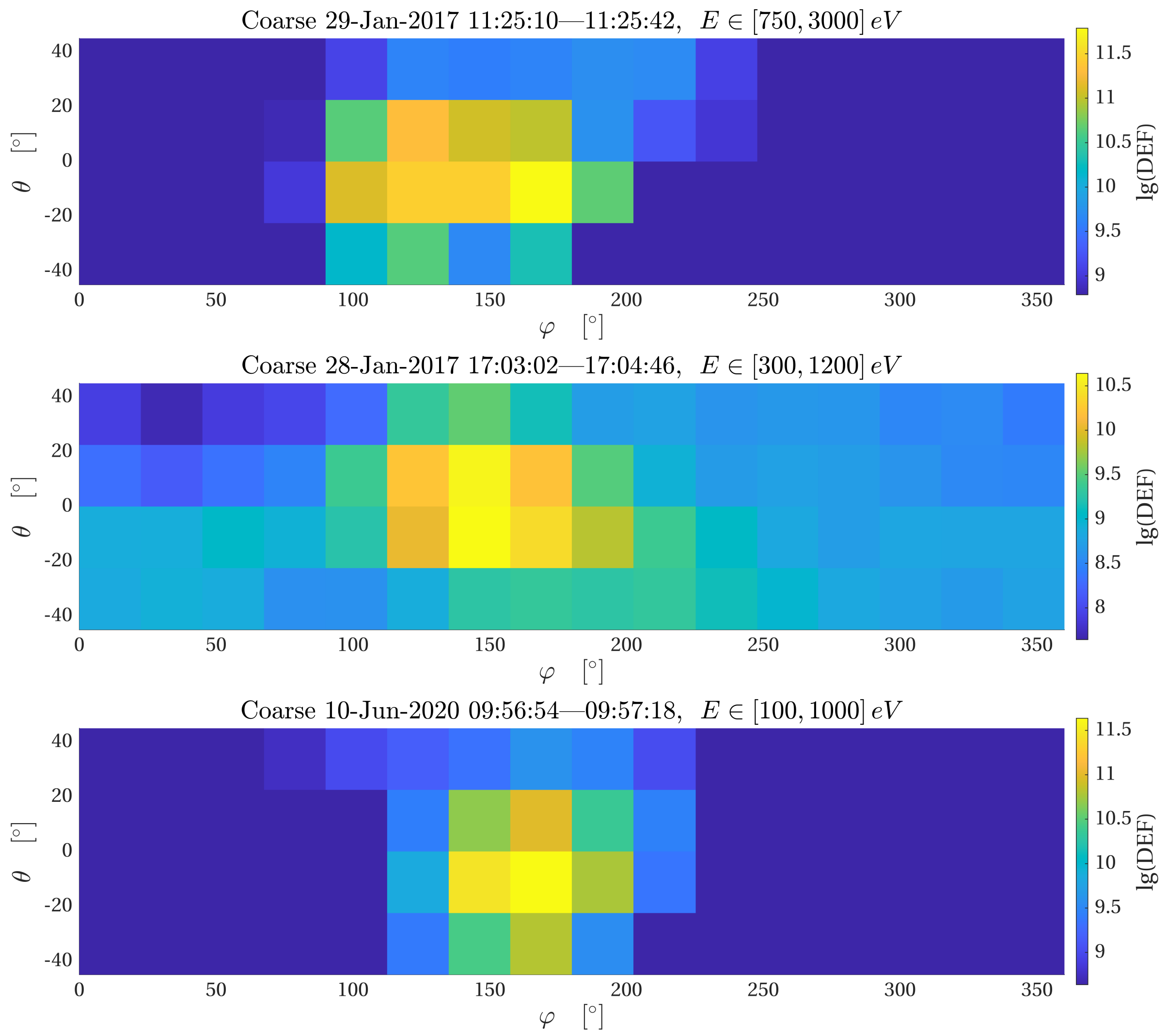

Figure S3: **Field of view.** Differential energy flux for the ions of the SWIA coarse integrated over the energy ranges that are indicated above each panel and averaged over the likewise indicated intervals.
